# Supplementary material for: The Effectiveness of Nintedanib in Patients with Idiopathic Pulmonary Fibrosis, Familial Pulmonary Fibrosis and Progressive Fibrosing Interstitial Lung Diseases: A Real-World Study
Source: Biomedicines. 2022 Aug 15;10(8):1973. doi: 10.3390/biomedicines10081973 (PMC9405731; doi:10.3390/biomedicines10081973)
Supplement: Supplementary file 1 [file biomedicines-10-01973-s001.zip › biomedicines-1844561-supplementary.pdf]

biomedicines

MDPI

The effectiveness of nintedanib in patients with idiopathic pulmonary fibrosis, familial pulmonary fibrosis and progressive fibrosing interstitial lung diseases: a real-world study

Supplementary Materials

Table S1. Comparison of annual FVC and DLCO decline rate in the entire study population. *: paired comparison.

Parameters	Pre-treatment	12-months	24-months	36 months	48 months
N°	37	88	49	24	10
**Δ FVC ml**	- 256 ± 352	-123.7 ± 297	-156.7 ± 285.8	-121.5 ± 254.7	-164.7 ± 200
p-value		p=0.0374*	p=0.5589	p=0.6598	p=0.2259
mean difference (95% CI)		-133.1 (-258.4 to 7.803)			
**ΔDLCO ml/min/mmHg**	-0.82 ± 1.75	-0.27 ± 1.6	-0.3 ± 1.1	-0.42 ± 0.9	-0.51 ± 2.4
p-value		p=0.0428*	p=0.7812	p=0.8856	p=0.4967
mean difference (95% CI)		-0.55.1 (-1.87 to 0.30)			
